# A Genetic Compensation Phenomenon and Global Gene Expression Changes in Sex-miR-2766-3p Knockout Strain of *Spodoptera exigua* Hübner (Lepidoptera: Noctuidae)

**DOI:** 10.3390/insects13111075

**Published:** 2022-11-21

**Authors:** Yayun Zuo, Zeyu Wang, Xuan Ren, Yakun Pei, Ahmed A. A. Aioub, Zhaonong Hu

**Affiliations:** 1 Institute of Pesticide Science, College of Plant Protection, Northwest A&F University, Yangling 712100, China; 2 Key Laboratory for Botanical Pesticide R & D of Shaanxi Province, Yangling 712100, China; 3 Plant Protection Department, Faculty of Agriculture, Zagazig University, Zagazig 44511, Egypt; 4 Key Laboratory of Crop Pest Integrated Pest Management on the Loess Plateau of Ministry of Agriculture, College of Plant Protection, Northwest A&F University, Yangling 712100, China; 5 State Key Laboratory of Crop Stress Biologyfor Arid Areas, Northwest A&F University, Yangling 712100, China

**Keywords:** microRNA, *CncC*, CRISPR/Cas9, beet armyworm, genetic robustness

## Abstract

**Simple Summary:**

miRNAs are endogenous, small, non-coding RNA species that can be crucial regulators in a variety of biological processes, such as development, immunity, and metamorphosis. Because one miRNA may regulate hundreds of targets, nearly all genes are expected to be regulated by miRNAs to some degree. Since this is the case, miRNA knockouts may show obvious symptoms, such as developmental defects and embryonic mortality. In this study, we reported that Sex-miR-2766-3p knockout in *S. exigua* was non-lethal, confirming the theory that miRNAs function redundantly with other miRNAs or other pathways.

**Abstract:**

MicroRNAs (miRNAs) drive the post-transcriptional repression of target mRNAs and play important roles in a variety of biological processes. miR-2766-3p is conserved and abundant in Lepidopteran species and may be involved in a variety of biological activities. In this study, Sex-miR-2766-3p was predicted to potentially bind to the 3′ untranslated region (UTR) of cap ‘n’ collar isoform C (CncC) in *Spodoptera exigua*, and Sex-miR-2766-3p was confirmed to regulate the expression of *SeCncC* through screening with a luciferase reporter system. Although CRISPR/Cas9 has been extensively utilized to examine insect gene function, studies of miRNA function are still relatively uncommon. Thus, we employed CRISPR/Cas9 to knock out Sex-miR-2766-3p from *S. exigua*. However, the expression of *SeCncC* was not significantly altered in the knockout strain (2766-KO) compared with that of the WHS strain. This result suggested that a miRNA knockout might lack phenotypes because of genetic robustness. Additionally, we used transcriptome analysis to examine how the global gene expression patterns of the Sex-miR-2766-3p knockout strain varied. RNA-seq data revealed 1746 upregulated and 2183 downregulated differentially expressed genes (DEGs) in the 2766-KO strain, which might be the result of Sex-miR-2766-3p loss or DNA lesions as the trigger for transcriptional adaptation. GO function classification and KEGG pathway analyses showed that these DEGs were enriched for terms related to binding, catalytic activity, metabolic process, and signal transduction. Our findings demonstrated that *S. exigua* could compensate for the missing Sex-miR-2766-3p by maintaining the expression of *SeCncC* by other pathways.

## 1. Introduction

MicroRNAs (miRNAs) are endogenous, non-coding, single-stranded RNAs that are 22 nucleotides in length [1]. miRNAs drive the post-transcriptional repression of target mRNAs and play key roles in the signaling pathways of development, cellular differentiation, proliferation, apoptosis, and oncogenesis that exist in a broad variety of vertebrates and invertebrates [2]. Since the first miRNA was identified from *Caenorhabditis elegans*, many endogenous miRNAs have been identified in a wide range of taxa, such as mammals, plants, and viruses [2,3,4]. Conserved and unique miRNAs in various species have been abundantly found and recognized, especially with the introduction of next-generation sequencing technology [5]. Thus far, a total of 38,589 mature miRNAs from 271 species have been discovered and submitted to the miRBase database (v22) [6]. Numerous miRNAs from different insect species have been discovered using next-generation sequencing technologies [7]. Recently, a study showed that nine specific miRNAs (including miR-2766-3p) in six insects had homologs, indicating that these miRNAs had conserved functions across Lepidopteran species [8]. In *Bombyx mori*, *Helicoverpa armigera*, and *Spodoptera litura*, miR-2766-3p was predicted to potentially bind to the 3′ untranslated region (UTR) of *Hsp90, PBANR*, and *TCT* [8]. Shen et al. (2022) found that injecting miR-2766-3p agomirs decreased the mRNA and protein levels of *HaTH* and led to abnormal pupation in *H. armigera* larvae, because the miR-2766-3p targeted the 3’ UTR of *HaTH* to post-transcriptionally regulate *HaTH* function [9]. In *Spodoptera frugiperda,* the expression of miR-2766-3p considerably increased after a treatment with spinetoram and emamectin benzoate, indicating that miR-2766-3p likely regulated pesticide tolerance [10].

The type II clustered regularly interspersed short palindromic repeat (CRISPR)/associated protein-9 nuclease (Cas9) system is a potent technique for manipulating a genome [11,12]. After a guide RNA (sgRNA) attaches to the target DNA fragment, the CRISPR/Cas9 system can cause double-strand breaks (DSBs), which are typically repaired by either non-homologous end joining (NHEJ) or homology-directed repair (HDR). Gene insertions or deletions may be created through NHEJ or HDR [13,14]. There are now reports that many insects have minor insertions and deletions caused by the CRISPR/Cas9 system [15]. It has also been used to knock in a mutation [16,17] and knock out a large genomic fragment [18] or a gene cluster [19] in *S. exigua* through a dual sgRNA system. Consequently, the CRISPR/Cas9 system may be a useful tool to investigate the functions of miRNAs.

*S. exigua* is a worldwide pest that causes significant crop losses [20] and has developed resistance to a wide range of insecticides [21]. The cap ‘n’ collar isoform C (CncC) plays a vital role in defending the organism against xenobiotic or oxidative stress by regulating many detoxification enzyme genes [22]. In this study, a miRNA, Sex-miR-2766-3p, was identified to potentially bind to the 3′ UTR of *CncC*. Then, the luciferase reporter assay in vitro revealed that Sex-miR-2766-3p could regulate *SeCncC* expression. Further, we deleted a 250 bp genomic fragment covering the partial promotor region and DNA template of pri-miRNA transcription in *S. exigua* through CRISPR/Cas9. However, the expression of *SeCncC* was not dramatically altered in the knockout strain (2766-KO). In addition, the variability of global gene expression patterns in the 2766-KO strain was investigated using transcriptome analysis. This work offers a fresh perspective and method for investigating the role of insect miRNAs. Our findings also suggest that genome editing may cause global changes in gene expression and genetic compensation.

## 2. Experimental Procedures

### 2.1. Insects *and Cell Lines*

The susceptible strain (WHS), gathered in 1998 from Wuhan in the province of Hubei (China), was received from the laboratory of Insect Molecular Toxicology at Nanjing Agricultural University and kept in a laboratory without exposure to insecticides. The 2766-KO strain was homozygous and was produced by knocking out miRNA-2766.

Dulbecco’s Modified Eagle Medium (DMEM) (Gibco), supplemented with 10% (vol/vol) fetal bovine serum (FBS) (Gibco), was used to cultivate HEK293T cells at 37 °C in an incubator with 5% CO_2_.

### 2.2. Dual-Luciferase Reporter (DLR) Assay

The primers used for the wild-type (WT) or mutated (MT) target sequence of *SeCncC* are listed in **Table 1**. The wild-type (WT) or mutated (MT) target sequence of *SeCncC* was made by PCR with PrimeSTAR Max DNA Polymerase (Takara, Dalian, China) with no template and two long primers in 50 mL reaction volumes. PCR was performed at 98 °C for 30 s; in 35 cycles of 98 °C for 10 s, 60 °C for 30 s, and 72 °C for 20 s; at 72 °C for 10 min; and at 12 °C for 1 min. Then, the PCR products were purified with a PCR purification kit (Qiagen, Hilden, Germany). Subsequently, the wild-type (WT) or mutated (MT) target sequence of *SeCncC* was cloned and inserted into the pmirGLO plasmid (Promega, Madison, WI, USA) via the XhoI restriction site using a homologous recombination kit (Vazyme, Nanjing, China) to construct the luciferase reporter plasmids pmirGLO-WT and pmirGLO-MT, respectively. For high transfection efficiency and low background expression of targets, the mammalian HEK293T cell line was used for the DLR assay. The HEK293T cells were cultured in a 96-well plate and transfected with the reporter plasmids and miRNA mimic or negative control of mimic (NC mimic), using GP-transfect-Mate (Gene Pharma, Shanghai, China) according to the manufacturer’s instructions. Each well contained 1 μg plasmid and 120 μM miRNA mimic. Luciferase assays were performed by using the Dual-Glo^®^ Luciferase Assay System (Promega) 48 h post-transfection. Normalized firefly luciferase activity (firefly luciferase activity/Renilla luciferase activity) was compared with the control groups. For each transfection, the luciferase activity was averaged from the results of three replicates.

### 2.3. sgRNA Production

The primers for the synthetic guide RNA (sgRNA) templates are shown in Table 1. The guide RNAs were synthesized according to the instructions using the GeneArt Precision gRNA Synthesis Kit (Thermo Fisher, Shanghai, China). The PCR products were purified with a PCR purification kit (Omega Bio-Tek, USA). The GeneArt Precision gRNA Synthesis Kit (Thermo Fisher, Shanghai, China) was used to perform in vitro transcription of the sgRNAs, and the sgRNAs were purified in accordance with the manufacturer’s instructions. After that, sgRNAs were diluted with nuclease-free water to ~2 μg/μL and kept at −80 °C.

### 2.4. Embryo Microinjection

The eggs of the WHS strain (newly laid within two hours) on A4 paper were collected and washed with 1% (*v*/*v*) sodium hypochlorite solution and then rinsed twice with distilled water. The eggs were then adhered to a microscope slide using double-sided adhesive tape [16]. The eggs were injected with a mixture containing Cas9 protein (100 ng/μL, Thermo Fisher Scientific, Shanghai, China), sgRNA1 (300 ng/μL), and sgRNA2 (300 ng/μL) using a PV820 Pneumatic PicoPump (World Precision Instruments Inc., New Haven, CT, USA). The injected eggs were kept at 26 ± 1 °C and 60 ± 10% relative humidity (RH) until hatching.

### 2.5. CRISPR/Cas9 Knockout of Sex-miR-2766-3p

Primers (JC-miRNAF/JC-miRNAF) were designed and used to amplify gDNA to identify the mutations caused by CRISPR/Cas9. We obtained gDNA from individuals using the M5 Universal DNA Mini Kit (Mei5 Biotechnology, Co., Ltd., Beijing, China). The PCRs contained 12.5 µL of 2×M5 HiPer Taq PCR mix (with blue dye) (Mei5 Biotechnology, Co., Ltd., Beijing, China), 1 µL of each 10 µM sense and antisense primer (Table 1), 1 µL gDNA, and 9.5 µL ddH_2_O. PCR was performed at 94 °C for 3 min; in 32 cycles of 94 °C for 20 s, 56 °C for 20 s, and 72 °C for 2 min; at 72 °C for 5 min; and at 12 °C ∞. Five microliters of each PCR were separated on 1.25% agarose gels stained with ethidium bromide. The DNA bands were gel-purified, TA-cloned into the pClone007 blunt vector (Tsingke BioTech, Beijing, China), and Sanger-sequenced by Tsingke BioTech (Xi’an, China).

**Figure 1:**
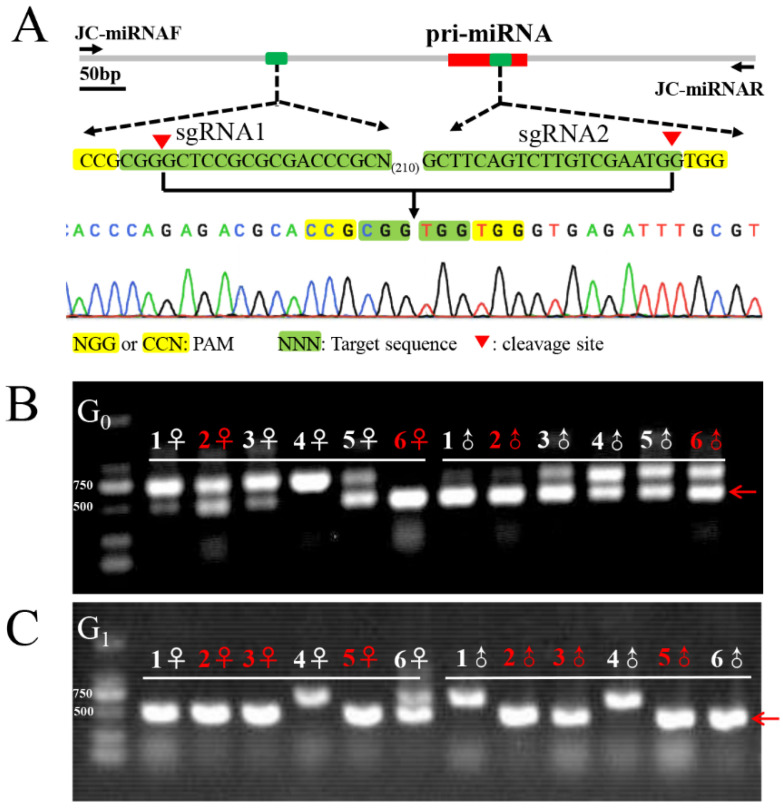
CRISPR/Cas9-induced genomic mutagenesis. Schematic representation of the single guide RNA (sgRNA) targeting sites (**A**). The green rectangle indicates the location of the sgRNAs target sequence; The red rectangle indicates the position of pri-miRNA sequence. Amplification by PCR to determine the genomic mutagenesis of individual moths in G_0_ (**B**). DNA fragments were amplified by PCR from the genomic DNA of the G_0_ moths after oviposition. The red arrow indicates deletion events in the genomic region between the two target sites across this region. The red number on the top indicates the single-pair number of G_0_ moths that were selected to produce G_1_ offspring. (**C**) Amplification by PCR to determine the genotype of individual moths in G_1_. The red number indicates female or male individuals, which are homozygous, that were selected to produce G_2_ offspring.

The G_0_ moths were sibling-crossed with one another to produce 24 single pairs, which were then used to construct homozygous strains for the Cas9-induced mutations. Each moth from a single pair was used for gDNA extraction after it laid eggs. To identify the genotypes and choose which lines to keep, PCR products flanking the two sgRNA target sites were amplified as described previously (Figure 1B). Only progeny for the desired positive mutations were maintained (Figure 1B). When they reached adulthood, the progeny (G_1_) from the positive single couples were retained for heritable detection and homozygous strain selection (Figure 1C).

### 2.6. RNA Sequencing

Six samples (2766-KO and WHS strains containing three biological replicates each, and each biological replicate containing ten third instar larvae) were sequenced on a BGISEQ-500 platform (Tsingke Biotechnology Co., Ltd., Xi’an, China) using RNA-seq technology as follows: (i) 1 μg total RNA was extracted from the WHS and 2766-KO strains using TRIzol^®^ reagent (Invitrogen, Carlsbad, CA, USA) and treated with DNase I (Takara, Dalian, China) to remove the genomic Oligo(dT)-attached magnetic beads that were used to purified mRNA; (ii) the purified mRNA was fragmented into small pieces with fragment buffer at an appropriate temperature and generated first-strand cDNA using random hexamer-primed reverse transcription incubated at 42 °C for 15 min, followed by double-strand cDNA (dscDNA) synthesis; (iii) the dscDNAs were treated by a traditional process, including end repairing with phosphate at the 5′ end, stickiness ‘A’ at the 3′ end, and ligation and adaptor with stickiness ‘T’ at the 3′ end; (iv) two specific primers were used to amplify the ligation product; (v) the double-stranded PCR products from the previous step were heated until they were denatured and circularized by the splint oligo sequence to obtain the final library; and (vi) the final library was amplified with phi29 to make a DNA nanoball (DNB), which had more than 300 copies of one molecule. DNBs were loaded into the patterned nanoarray, and 150 pair-end base reads were generated on the DNBSEQ-T7 platform (Huada Zhizao, Shenzhen, China).

The raw reads were filtered via SOAPnuke (v1.4.0) [23] by removing the reads containing adapters or poly-N or that were low quality, and then clean reads were obtained. Trinity (v2.0.6) [24] was used to assemble the clean reads, and Tgicl (v2.0.6) [25] was used to perform clustering and eliminate redundant data in the assembled transcripts to obtain unique genes. The assembled transcripts were processed for further expression analysis and functional annotation. The clean reads were mapped to the assembled unique genes by Bowtie2 (v2.2.5) [26], and the expression levels of the genes were calculated by RSEM (v1.2.8) [27] and normalized to FPKM (fragments per kilobase of transcript per million mapped reads). The functional annotation of the genes was achieved by mapping the genes to different databases (NT, NR, KOG, and KEGG) using the software BLAST (v2.2.23) [28]. GO annotation was performed using Blast2GO (v 2.5.0) with NR annotations. PossionDis [29] was used to detect differentially expressed genes (DEGs), and DEGs with |log_2_(fold change)| > 2 and a false discovery rate (FDR) < 0.001 were considered to be significantly differentially expressed genes. GO enrichment analysis and KEGG enrichment analysis were performed using Phyper, a function of R. The significance levels of terms and pathways were corrected by the Q value with a rigorous threshold (Q value < 0.05). The potential Sex-miR-2766-3p binding genes in upregulated genes were predicted using RNAhybrid (https://bibiserv.cebitec.uni-bielefeld.de/rnahybrid?id=rnahybrid_view_submission accessed on 8 November 2022) [30].

### 2.7. RNA Extraction and Quantitative RT-PCR

The total RNA from the third instar larvae of WHS or the 2766-KO strain of *S. exigua* was extracted using TRIzol reagent (Invitrogen, Carlsbad, CA, USA) and then treated with DNase I (Takara, Dalian, China) to prevent genomic DNA contamination. Small RNAs were isolated from the third instar larvae of the WHS or 2766-KO strain using the miRcute miRNA isolation kit (Tiangen, Beijing, China). RNA extraction was performed according to the manufacturer’s instructions. For mRNA, the PrimeScript™ 1st Strand cDNA Synthesis Kit (Takara, Dalian, China) was used to generate cDNA; 2xNovoStar^®^ SYBR qPCR SuperMix Plus was used to perform real-time PCR; and β-actin and glyceraldehyde-3-phosphate dehydrogenase (GAPDH) were utilized as internal controls. Five upregulated genes and 4 downregulated genes (primers were listed in Supplementary Table S1) were randomly chosen for RT-qPCR examination to confirm the validity of the DEG findings. For miRNA, the miRcute Plus miRNA First-Strand cDNA Kit (Tiangen, Beijing, China) was used to generate cDNA; the miRcute Plus miRNA qPCR Kit (SYBR Green) (Tiangen, Beijing, China) was used to perform real-time PCR; and U6 small nuclear RNA (snRNA) was utilized as an internal control. Real-time PCR was performed using a 240 LightCycler 480 II system (Roche Diagnostics, Mannheim, Germany). All reactions were performed in triplicate. The primers used in this study were synthesized by Tsingke Biotechnology Co., Ltd. (Xi’an, China). The reverse primer for miRNA was supplied by the miRcute Plus miRNA qPCR Kit (SYBR Green). The 2^−ΔΔCt^ method was used to calculate the relative expression of RNAs [31]. A T test at a significance level of 0.05 was used to test whether the expression level of the genes was significantly different between the WHS and 2766-KO strains using SPSS 20.0 software (SPSS Inc., Chicago, IL, USA).

## 3. Results

### 3.1. Sex-miR-2766-3p Regulates the Expression of SeCncC In Vitro

**Figure 2:**
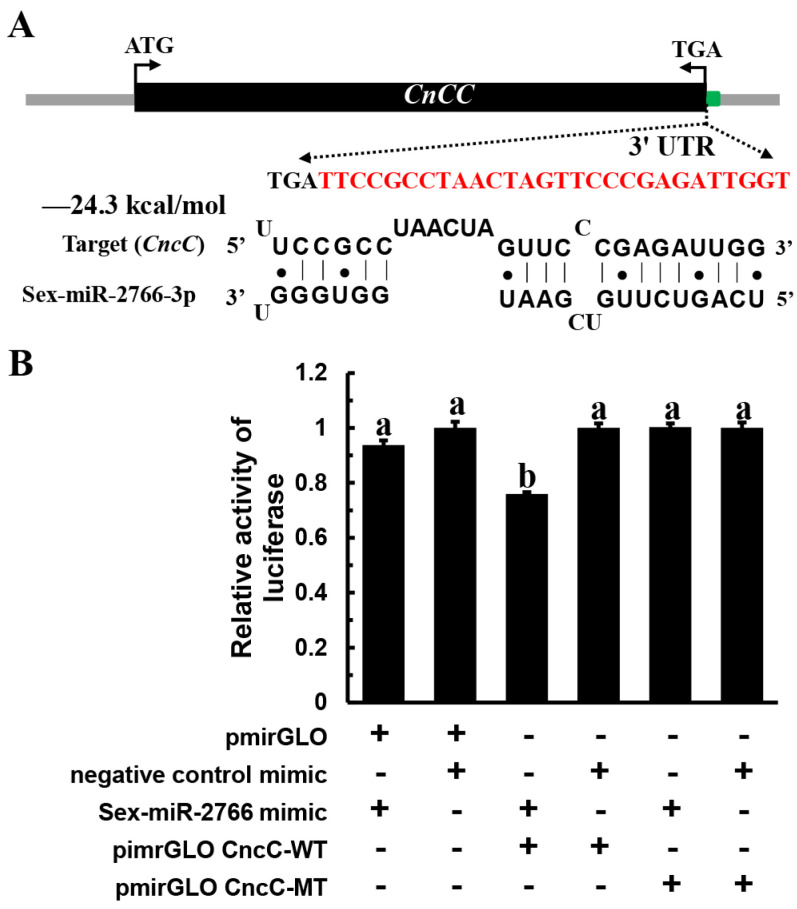
Verification of the interaction between Sex-miR2766 and *SeCncC* using a dual fluorescent reporter system. (**A**) Predicted target site (red bases) of Sex-miR-2766-3p in the 3′ UTR of *SeCncC*. (**B**) Luciferase reporter assays were performed to validate the interaction of Sex-miR-2766-3p with *SeCncC* in vitro. The mutated nucleotides are shown in italics. Data are presented as the means ± standard errors. The data were analyzed using analysis of variance followed by Tukey’s multiple comparisons, and the treatments designated with different letters were significantly different from each other at *p <* 0.05.

We used dual-luciferase reporter assays by co-transfecting a Sex-miR-2766-3p mimic and a recombinant pmirGLO vector with 200 bp cDNA fragments of *SeCncC* containing the predicted miRNA-binding sites into HEK293T cells to determine whether Sex-miR-2766-3p could act on the predicted binding sites and regulate the expression of *SeCncC* in vitro. The luciferase activity decreased by 24% (48 h) when the Sex-miR-2766-3p mimic was co-transfected with the pmirGLO-WT vector (wild-type) into HEK293T cells compared with the NC mimic control (Figure 2B). However, compared with the negative controls, the co-transfection of the Sex-miR-2766-3p mimic and recombinant pmirGLO-MT vector (mutant type) had no impact on the luciferase activity. The luciferase assay findings revealed that the predicted miRNA binding sites in the *SeCncC* 3′ UTR were functional and might be targeted by Sex-miR-2766-3p in HEK293 cells.

### 3.2. CRISPR/Cas9-Mediated Knockout of Sex-miRNA-2766-3p

**Figure 3:**
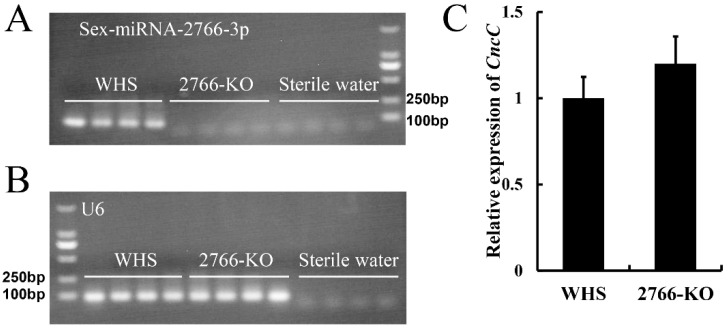
The examined expression of Sex-2766-miRNA-3p (**A**), U6 (**B**) and *CncC* (**C**) at transcriptional level between WHS and 2766-KO strains.

To further study this possibility, we knocked out Sex-miRNA-2766 in *S. exigua*. Seventy eggs (16.9%) of the 414 total implanted eggs hatched. A total of 58 larvae (82.9%) pupated. After oviposition, the genotype of 12 single pairs of G_0_ moths (high number of eggs) was determined by PCR. To create G_1_, the positive single pairs #2 and #6 were used. (Figure 1A). After the single pairs laid eggs, sequencing of the males and females from 12 single pairs of G_1_ moths (high number of eggs) revealed that the single pairs #2, #3, and #5 were homozygous for Sex-miRNA-2766 knockout (Figure 1B). Then, the progenies of single pairs #2, #3, and #5 were pooled to produce G_3_. The genotypes of 16 randomly chosen second instar larvae from G_3_ were homozygous, demonstrating that the Sex-miRNA-2766 knockout strain (abbreviated 2766-KO) had been built. The genome-edited 243-bp deletion included a partial promoter sequence and a partial precursor sequence of Sex-miR-2766-3p, resulting in the loss of Sex-miR-2766-3p. In addition, the transcription level showed that Sex-miR-2766-3p was not found in the 2766-KO strain (Figure 3A).

### 3.3. Effect of Sex-miR-2766-3p Knockout on the Expression of the SeCncC Gene

The RNA-seq data showed that *SeCncC* with log_2_(fold change) was 1.79 (<2, Supplementary Table S2), which was not significant. To further analyze the effect of the Sex-miR-2766-3p knockout on the expression of *SeCncC*, we analyzed the expression of *SeCncC* in the WHS strain and 2766-KO strain by RT-PCR. The results also indicated that *SeCncC* was not significantly changed after loss of miR-2766-3p (*t* test, *t* = 1.72, *df* = 2, *p* = 0.16).

### 3.4. Effect of Sex-miR-2766-3p Knockout on Global Gene Expression

**Figure 4:**
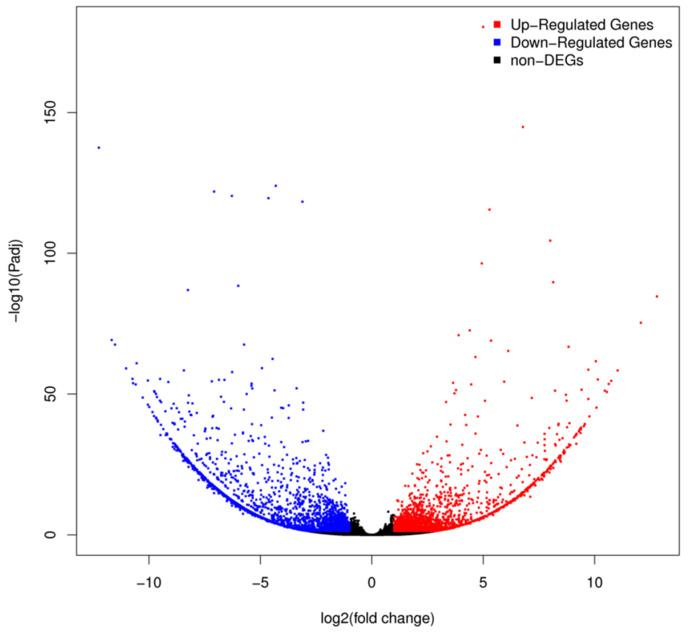
Differentially expressed genes between the 2766-KO strain and WHS train. X-axis shows the log_2_ (fold change), and Y-axis shows the −log^10^ (*p*-values). The higher the value is on the Y-axis, the lower the corresponding adjusted *p*-value is. All dots represent a fold change ≥ 2 and *p*-value ≤ 0.001. Red dots indicate upregulated DEGs. Blue dots indicate downregulated DEGs. Black dots indicate non-regulated genes.

We obtained ~6.87 Gb of data on average for each sample. After filtering, 47.24 M, 45.14 M, and 44.37 M clean reads were obtained from the WHS strain, and 45.78 M, 45.04 M, and 47.33 M clean reads were obtained from the 2766-KO strain (Supplementary Table S3). The Q20 and Q30 ratios of all the samples were ~98% and ~94%, respectively, according to the quality of data evaluation, while the GC counts varied from 46.87% to 47.52% (Supplementary Table S3). The 45,795 unigenes created from these clean readings were assembled. Compared with that of WHS, the miRNA-2766-KO knockout strain had 1746 DEGs that were upregulated and 2183 DEGs that were downregulated, according to transcriptome analysis (Supplementary Table S2). We predicted 188 potential Sex-miR-2766-3p binding genes in upregulated genes using RNAhybrid (Supplementary Table S4). Figure 4 depicts a volcano graph showing the outcomes when |log_2_(fold change)| is >2 and a *p* value ≤ 0.001.

**Figure 5:**
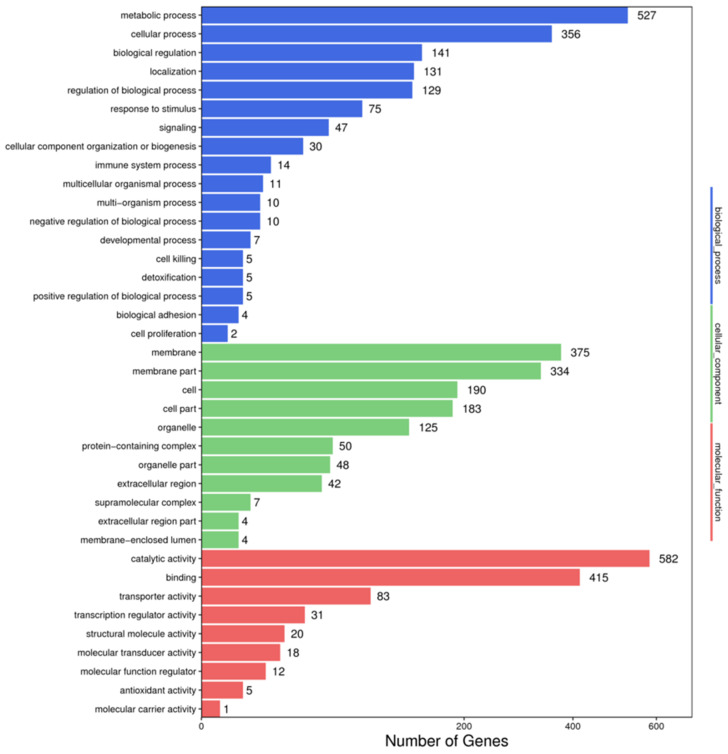
GO functional classification of DEGs from the 2766-KO knockout strain. X-axis indicates the number of DEGs. Y-axis represents GO terms.

**Figure 6:**
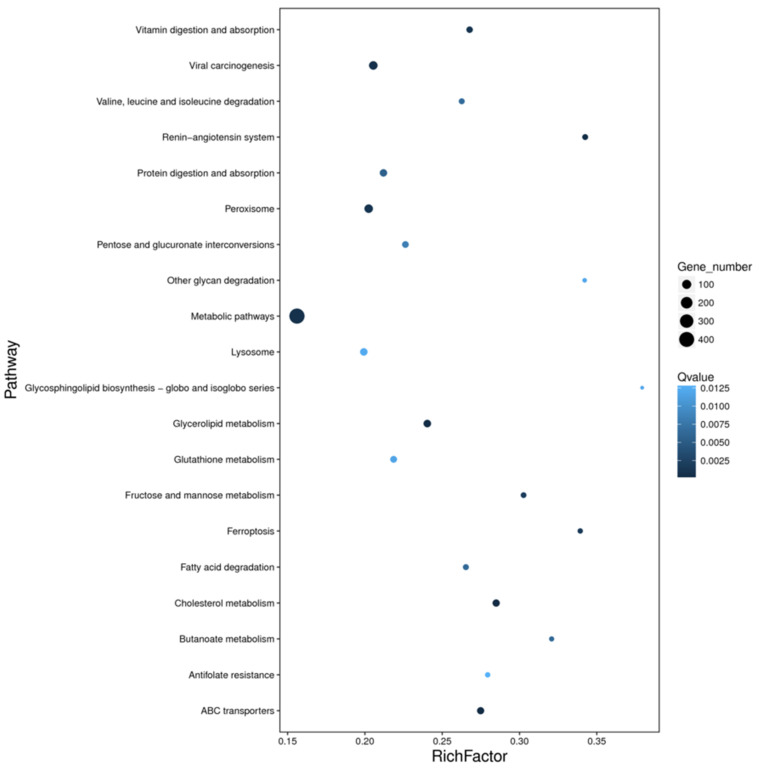
Scatter plot of the KEGG enrichment of differential genes. The vertical axis represents the name of the pathway, the horizontal axis indicates the rich factor, the size of the dot represents the number of differentially expressed genes in a given pathway, and the colors of the dots correspond to different Q value ranges.

The GO analysis indicated that DEGs were mainly summarized into three categories, including 1509 biological process (BP) terms, 1167 molecular function (MF) terms, and 1362 cellular component (CC) terms (Figure 5 and Supplementary Table S5). Among the molecular functions, differentially expressed genes accounted for the highest proportion in the two functional subcategories of catalytic activity and binding. In the biological process category, differentially expressed genes accounted for the highest proportion in the two functional subcategories of metabolic process and cellular process. Meanwhile, the KEGG pathway has seven main categories, including cellular processes, environmental information processing, genetic information processing, human disease, metabolism, and organismal systems (Figure 6 and Supplementary Table S6). KEGG annotation indicated that the metabolism pathway was the most prominent branch, which had 1235 DEGs. Among them, the “global and overview maps” branch of the metabolism pathway was the most prominent branch, and it had 423 DEGs. “Signal transduction” of the environmental information processing pathway was the second most prominent branch with 372 DEGs. These results indicated that Sex-miRNA-2766-3p knockout affects global gene expression, especially in catalysis and metabolism.

**Figure 7:**
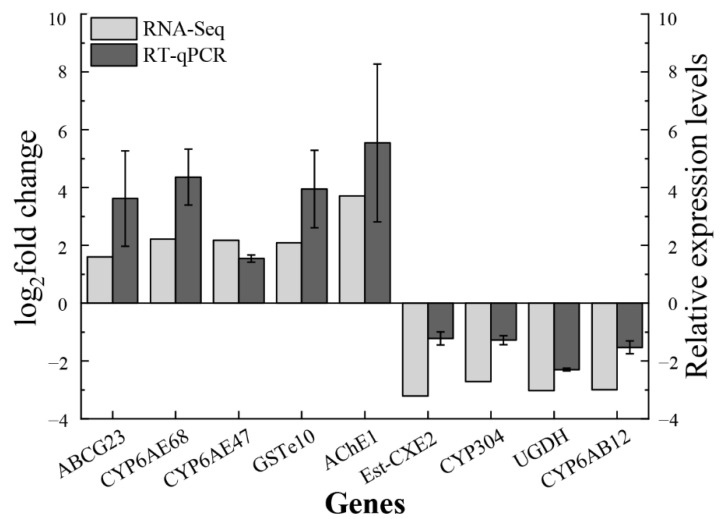
RT-qPCR validation of DGE results. Five upregulated genes and four downregulated genes were identified by RT-qPCR. The left y-axis represents the log_2_ fold change by RNA-seq, and the right y-axis represents the relative expression level by RT-qPCR.

Five upregulated genes and four downregulated genes were randomly chosen for RT-qPCR examination to confirm the validity of the DEG findings. The outcomes demonstrated that the RT-qPCR results and the expression of five upregulated genes and five downregulated genes in DEG data were both consistent (Figure 7).

## 4. Discussion

miRNAs are endogenous, small, non-coding RNA species that directly target the 3′ UTR of mRNA targets to regulate post-transcriptional gene expression [32]. miRNAs can be crucial regulators in a variety of biological processes, such as development, immunity, and metamorphosis [33]. One miRNA may regulate hundreds of targets, according to computational assessments, microarrays, proteomics methods, and high-throughput sequencing analysis. In mammalian genomes, nearly all genes are expected to be regulated by miRNAs to some degree, according to the available 500–800 miRNAs [33]. Since this is the case, miRNA knockouts may show obvious symptoms such as developmental defects and embryonic mortality. In this study, we reported that Sex-miR-2766-3p knockout in *S. exigua* was non-lethal, confirming the theory that miRNAs function redundantly with other miRNAs or other pathways [34].

The luciferase reporter system is frequently employed to confirm the relationship between miRNAs and genes. In this study, using dual-luciferase reporter plasmids to transfect HEK293T cells, we validated that Sex-miR-2766-3p regulates the expression of *SeCncC*. The luciferase activity decreased by 24% when the miR-2766-3p mimic was cotransfected into HEK293T cells together with the pmirGLO-WT vector (wild-type) as opposed to the NC mimic control. The suppression efficiency of Sex-miR-2766-3p on *SeCncC* in *S. exigua* was similar to the suppression efficiency of Sex-miR-2766-3p on *Hsp90* in *H. armigera*, *S. litura*, or *B. mori* (approximately 20%) [8] but lower than the suppression efficiency of miR-2766-3p on *HaTH* (47%) [9]. The suppression efficiency of miRNA on the target gene may be related to minimum free energy (mfe) of hybridization between the miRNA and the mRNA of the target sequence. In this study, the minimum free energy (mfe) of hybridization between Sex-miR-2766-3p and 3′ UTR of *SeCncC* was −24.3 kcal/mol (Figure 2A), which was higher than the mfe of hybridization between miR-2766-3p and 3′ UTR of *HaTH* (−27.3 kcal/mol). The lower mfe indicates greater stability of the hybridization, which may be the reason for the low suppression efficiency in this study.

However, the expression of *SeCncC* was not significantly changed in the knockout strain (2766-KO) compared with that of the wild-type WHS strain. This phenomenon may be the result of genetic robustness. According to earlier research, living organisms have the capacity to protect themselves against harmful genome mutations and retain their fitness and viability in the face of genetic changes, such as the loss of functional genes [35]. Genetic robustness can cause the results of gene knockout or knockdown in vivo to be misinterpreted; for example, the phenotypes of knockout strains may not accurately or completely represent the removed genes [35]. This phenomenon has been found in many mouse [36,37], zebrafish [38], insect [39], and plant mutants [40,41]. The loss of one gene may be compensated by another with overlapping functions and expression patterns, leading to genetic resilience, as observed for numerous mutants in a variety of model species [35]. Because many miRNAs collaborate with other miRNAs or other pathways, removing one of the members would not result in a reduction in target gene expression below the level required to produce phenotypic effects [33]. In addition, cellular networks, including metabolic, signaling, and transcriptional networks, closely control gene expression. The perturbation of a post-transcriptional regulator network may maintain cellular wellness by altering the expression of other genes via the same network or other networks. Thus, the disruption of a gene network due to the CRISPR/Cas9-mediated deletion of Sex-miR-2766-3p may trigger a genetic robustness response to preserve the equilibrium of *SeCncC* expression in *S. exigua*.

The knockout of Sex-miR-2766-3p did not affect *SeCncC* expression but did affect the expression of a large number of genes involved in binding, catalytic activity, metabolic processes, and signal transduction. We attributed the DEGs to the possibility that Sex-miR-2766-3p loss or DNA lesion may have caused the transcriptional adaptation response. Sex-miR-2766-3p was verified to target the 3′ UTR of *HaTH* to post-transcriptionally regulate *HaTH* expression in *H. armigera* [9] and the 3′ UTR of *Hsp90* to post-transcriptionally regulate *Hsp90* expression in *H. armigera, S. litura,* and *B. mori* [8]. In addition, 188 potential Sex-miR-2766-3p-binding genes were predicted in upregulated genes using RNAhybrid, which might be directly impacted by the Sex-miR-2766-3p knockout. The other DEGs might be indirectly impacted by the Sex-miR-2766-3p knockout. However, these need to be verified further. Therefore, the loss of miR-2766-3p will affect the expression of its regulated genes, and the change in the expression of its regulated genes will lead to the change in the expression of upstream and downstream genes of its regulated genes. Furthermore, the CRISPR/Cas9 system introduces DSBs, which lead to DNA damage, chromatin reorganization, and decondensation [42]. The stress induced by DNA damage also leads to changes in interchromosomal interactions, leading to specific gene upregulation [35]. Chromatin reorganization may accompany changes in DNA looping and nuclear structure [43], potentially impacting gene expression. In brief, these findings imply that *S. exigua* rewires the genetic and cellular network to address the Sex-miR-2766-3p deletion caused by CRISPR/Cas9.

Our findings suggest that miRNA knockouts may lack phenotypes because of genetic robustness. In future studies based on genome-editing techniques for studying miRNA function, this situation needs to be considered.

## Figures and Tables

**Table 1:** Primers used in this study for the construction of the luciferase reporter vector, for gRNA DNA temple synthesis, and for detecting large fragment knockout.

**Primers**	Primer Sequence (5′–3′)	**Purposes**
3UTR-MT-F	aacgagctcgctagcctcgagCCACCCAATGAACCGTACTTCCGACGATGATATGGACAGAAAAGCCAAGAGCTACGACCAGTGAT*AGGCGG*TAACTA*CAAG*C*GCTCTACC*	For making the mutated (MT) target sequence of *CncC*
3UTR-MT-R	caggtcgactctagactcgagCAATTATTGCGTTGTCCAAGTCTTGTATGTGTGTATATGTATTA*GGTAGAGC*G*CTTG*TAGTTA*CCGCCT*ATCACTGGTCG	
3UTR-WT-F	aacgagctcgctagcctcgagCCACCCAATGAACCGTACTTCCGACGATGATATGGACAGAAAAGCCAAGAGCTACGACCAGTGATTCCGCCTAACTAGTTCCCGAGATGG	For making the wild-type (WT) target sequence of *CncC*
3UTR-WT-R	caggtcgactctagactcgagCAATTATTGCGTTGTCCAAGTCTTGTATGTGTGTATATGTATTACCATCTCGGGAACTAGTTAGGCGGAATCACTGGTCG	
sgRNAF1	**TAATACGACTCACTATA** GCGGGTCGCGCGGAGCCCG	For making the DNA template of sgRNA1
sgRNAR1	TTCTAGCTCTAAAACCGGGCTCCGCGCGACCCG	
sgRNAF2	**TAATACGACTCACTATA** GCTTCAGTCTTGTCGAATGG	For making the DNA template of sgRNA2
sgRNAR2	TTCTAGCTCTAAAACCCATTCGACAAGACTGAAG	
JC-miRNAF	CATGCTTACACAGTAGGAACGT	For detecting large fragment knockout
JC-miRNAR	GCGGAAGTTACTACACAAAGGG	
qmiR-2766F	AGTCTTGTCGAATGGTGGGT	For quantitative RT-PCR
qU6F	TTGGAACGATACAGAGAAGATTAGC	
qCncCF	ACAGAGCAATATTCCCAGTCCG	
qCncCR	AAGAACCACCATCTGACATGCT	
qGADPHF	AACATTTATCTCTACAACGCAATC	
qGADPHR	GTGACAACCACTCATCTATCTTC	
β-actinF	AGCGTGACATCAAGAGGACT	
β-actinR	CTCCATGTATGCCTGCTTCG	

Lowercase letters indicated homologous arms of pmirGLO vector. Double-underlined indicated binding target sites of Sex-miR-2766-3p, and italic bases are mutated bases. Bold sequences indicated the T7 adaptor. The sequences targeted by sgRNA are underlined.

## Data Availability

Data is contained within the article or Supplementary Material.

## Supplementary Materials

The following supporting information can be downloaded at: https://www.mdpi.com/article/10.3390/insects13111075/s1, Table S1: Differentially expressed genes and their primer sequences used in RT-qPCR. Table S2: 2766-KO vs WHS DEGs. Table S3: Overview of the transcriptome sequencing data from the WHS and 2766-KO strain larvae. Table S4: The potential Sex-miR-2766-3p binding genes in up-regulated genes of DEGs. Table S5: 2766-KO vs WHS DEGs GO enrichment. Table S6: 2766-KO vs WHS DEGs KEGG enrichment.

## References

[1] Bartel D.P. (2004). MicroRNAs: Genomics, biogenesis, mechanism, and function. Cell.

[2] Bushati N., Cohen S.M. (2007). microRNA functions. Annu. Rev. Cell Dev. Biol..

[3] Lee R.C., Feinbaum R.L., Ambros V. (1993). The C. elegans heterochronic gen lin-4 encodes small RNAs with antisense complementarity to lin-14. Cell.

[4] He L., Hannon G.J. (2004). MicroRNAs: Small RNAs with a big role in gene regulation. Nat. Rev. Genet..

[5] Yi S., Gao Z.X., Zhao H., Zeng C., Luo W., Chen B., Wang W.-M. (2013). Identification and characterization of microRNAs involved in growth of blunt snout bream (*Megalobrama amblycephala*) by Solexa sequencing. BMC Genom..

[6] Kozomara A., Birgaoanu M., Griffiths-Jones S. (2019). miRBase: From microRNA sequences to function. Nucleic Acids Res..

[7] Zhang X., Zheng Y., Jagadeeswaran G., Ren R., Sunkar R., Jiang H. (2012). Identification and developmental profiling of conserved and novel microRNAs in *Manduca sexta*. Insect Biochem. Mol..

[8] Ge X., Zhang Y., Jiang J., Zhong Y., Yang X., Li Z., Huang Y., Tan A. (2013). Identification of microRNAs in *Helicoverpa armigera* and *Spodoptera litura* based on deep sequencing and homology analysis. Int. J. Biol. Sci..

[9] Shen Z.J., Zhu F., Liu Y.J., Li Z., Moural T.W., Liu X.M., Liu X. (2022). MicroRNAs miR-14 and miR-2766 regulate tyrosine hydroxylase to control larval-pupal metamorphosis in *Helicoverpa armigera*. Pest Manag. Sci..

[10] Yang Y., Zhang Y., Wang A., Duan A., Xue C., Wang K., Zhao M., Zhang J. (2022). Four MicroRNAs, miR-13b-3p, miR-278-5p, miR-10483-5p, and miR-10485-5p, mediate insecticide tolerance in *Spodoptera frugiperda*. Front. Genet..

[11] Hsu P.D., Lander E.S., Zhang F. (2014). Development and applications of CRISPR-Cas9 for genome engineering. Cell.

[12] Sander J.D., Joung J.K. (2014). CRISPR-Cas systems for editing, regulating and targeting genomes. Nat. Biotechnol..

[13] Beumer K.J., Trautman J.K., Bozas A., Liu J.L., Rutter J., Gall J.G., Carroll D. (2008). Efficient gene targeting in *Drosophila* by direct embryo injection with zincfinger nucleases. Proc. Natl. Acad. Sci. USA.

[14] Beumer K.J., Trautman J.K., Mukherjee K., Carroll D. (2013). Donor DNA utilization during gene targeting with zinc-finger nucleases. G3-Genes. Genom. Genet..

[15] Li J.J., Shi Y., Wu J.N., Li H., Smagghe G., Liu T.X. (2021). CRISPR/Cas9 in lepidopteran insects: Progress, application and prospects. J. Insect Physiol..

[16] Zuo Y.Y., Wang H., Xu Y.J., Huang J.L., Wu S.W., Wu Y.D., Yang Y. (2017). CRISPR/Cas9 mediated G4946E substitution in the ryanodine receptor of *Spodoptera exigua* confers high levels of resistance to diamide insecticides. Insect Biochem. Molec. Biol..

[17] Zuo Y.Y., Xue Y.X., Wang Z.Y., Ren X., Aioub A.A., Wu Y.D., Yang Y.H., Hu Z.N. (2022). Knockin of the G275E mutation of the nicotinic acetylcholine receptor (nAChR) α6 confers high levels of resistance to spinosyns in *Spodoptera exigua*. Insect Sci..

[18] Zuo Y.Y., Xue Y.J., Lu W.J., Ma H.H., Chen M.H., Wu Y., Yang Y., Hu Z. (2020). Functional validation of nicotinic acetylcholine receptor (nAChR) α6 as a target of spinosyns in *Spodoptera exigua* utilizing the CRISPR/Cas9 system. Pest Manag. Sci..

[19] Zuo Y.Y., Shi Y., Zhang F., Guan F., Zhang J.P., Feyereisen R., Fabrick J.A., Yang Y., Wu Y. (2021). Genome mapping coupled with CRISPR gene editing reveals a P450 gene confers avermectin resistance in the beet armyworm. PLoS Genet..

[20] Berdegué M., Reitz S.R., Trumble J.T. (1998). Host plant selection and development in *Spodoptera exigua*: Do mother and offspring know best?. Entomol. Exp. Appl..

[21] Che W., Shi T., Wu Y., Yang Y. (2013). Insecticide resistance status of field populations of *Spodoptera exigua* (Lepidoptera: Noctuidae) from China. J. Econ. Entomol..

[22] Kalsi M., Palli S.R. (2017). Cap n collar transcription factor regulates multiple genes coding for proteins involved in insecticide detoxification in the red flour beetle, Tribolium castaneum. Insect Biochem. Mol. Biol..

[23] Chen Y., Chen Y., Shi C., Huang Z., Zhang Y., Li S., Li Y., Ye J., Yuxin C., Li Z. (2017). SOAPnuke: A MapReduce acceleration-supported software for integrated quality control and preprocessing of high-throughput sequencing data. Gigascience.

[24] Grabherr M.G., Haas B.J., Yassour M., Levin J.Z., Thompson D.A., Amit I., Adiconis X., Fan L., Raychowdhury R., Zeng Q. (2011). Trinity: Reconstructing a full-length transcriptome without a genome from RNA-Seq data. Nature Biotechnol..

[25] Pertea G., Huang X., Liang F., Antonescu V., Sultana R., Karamycheva S., Lee Y., White J., Cheung F., Parvizi B. (2003). TIGR Gene Indices clustering tools (TGICL): A software system for fast clustering of large EST datasets. Bioinformatics.

[26] Langmead B., Salzberg S.L. (2012). Fast gapped-read alignment with Bowtie 2. Nat. Methods.

[27] Li B., Dewey C.N. (2011). RSEM: Accurate transcript quantification from RNA-Seq data with or without a reference genome. BMC Bioinform..

[28] Miller W., Myers E.W., Lipman D.J. (2008). Blast (basic local alignment search tool). Encycl. Genet. Genom. Proteom. Inform..

[29] Audic S., Claverie J.M. (1997). The significance of digital gene expression profiles. Genome Res..

[30] Krüger J., Rehmsmeier M. (2006). RNAhybrid: microRNA target prediction easy, fast and flexible. Nucleic Acids Res..

[31] Livak K.J., Schmittgen T.D. (2001). Analysis of relative gene expression data using real-time quantitative PCR and the 2^− ΔΔCT^ method. Methods.

[32] Bartel D.P. (2009). MicroRNAs: Target recognition and regulatory functions. Cell.

[33] Ambros V. (2004). The functions of animal microRNAs. Nature.

[34] Park C.Y., Jeker L.T., Carver-Moore K., Oh A., Liu H.J., Cameron R., Richards H., Li Z., Adler D., Yoshinaga Y. (2012). A resource for the conditional ablation of microRNAs in the mouse. Cell Rep..

[35] El-Brolosy M.A., Stainier D.Y. (2017). Genetic compensation: A phenomenon in search of mechanisms. PLoS Genet..

[36] Meelad M.D., Kibibi G., Benjamin E.P., Hu Y., Styliani M., Albert W.C., Gao Q., Kim J., Choi S.W., Page D.C. (2011). Tet1 is dispensable for maintaining pluripotency and its loss is compatible with embryonic and postnatal development. Cell Stem. Cell..

[37] Monique N.O., Katherine H.S., Zhang Y., Anne-Cécile E.D., Shuyun R.J., Scott H., Academia E.C., Shah S.R., Morton J.F., Holstein C.A. (2013). The ribosomal protein Rpl22 controls ribosome composition by directly repressing expression of its own paralog, Rpl22l1. PLoS Genet..

[38] Rossi A., Kontarakis Z., Gerri C., Nolte H., Hölper S., Krüger M., Stainier D.Y. (2015). Genetic compensation induced by deleterious mutations but not gene knockdowns. Nature.

[39] Wang M., Zhang S., Shi Y., Yang Y.Y., Wu Y.D. (2020). Global gene expression changes induced by knockout of a protease gene cluster in *Helicoverpa armigera* with CRISPR/Cas9. J. Insect Physiol..

[40] Bouche N., Bouchez D. (2001). Arabidopsis gene knockout: Phenotypes wanted. Curr. Opin. Plant Biol..

[41] Rodriguez-Leal D., Xu C., Kwon C., Soyars C., Demesa-Arevalo E., Man J., Liu L., Lemmon Z.H., Jones D.S., Van Eck J. (2019). Evolution of buffering in a genetic circuit controlling plant stem cell proliferation. Nat. Genet..

[42] Pulecio J., Verma N., Mejía-Ramírez E., Huangfu D., Raya A. (2017). CRISPR/Cas9-based engineering of the epigenome. Cell Stem Cell.

[43] Chambeyron S., Bickmore W.A. (2004). Chromatin decondensation and nuclear reorganization of the HoxB locus upon induction of transcription. Genes Dev..

